# Supplementary material for: Diagnostic ability of Peptidase S8 gene in the Arthrodermataceae causing dermatophytoses: A metadata analysis
Source: PLoS One. 2024 Jul 9;19(7):e0306829. doi: 10.1371/journal.pone.0306829 (PMC11232979; doi:10.1371/journal.pone.0306829)
Supplement: S1 Table — (PDF) [file pone.0306829.s001.pdf]

Supplementary Table 1: The list of species included in this study with accession number and type of expressed subtilisin

Species	Subtilisin Subtype	Accession number
T. rubrum	SUB 1	XM_003236307, XM_047749566, EGD87150, KFL61190, KDB32093, KDB32094, EZG15174, EZG15175, EZF40365, EZF50872, EZG04634, AY343499, EZF16229, EZF16230, EZG04635, EZF93556, EZF83010, EZF40366, EZF83011, EZF50871, EZF93557, EZF61588, EZF61589
	SUB 2	XM_003235888, AY343500, EGD92042, EZG21620, KDB38432, EZG10961, EZF67982, EZF57408, EZF89230, EZG00132, XM_003231122, EZF46754, EZF27650
	SUB 3	XM_003236725, EGD87568, AY343501, EZG14624, KDB31555, EZF82478, EZF93159, EZG04304, EZF61008, EZF50416, EZF39787, EZF14297
	SUB 4	XM_003236843, XM_047749322, EGD85342, KFL60653, EZF13389, EZF13390, EZF39617, EZF50141, EZF60772, AY344481, EZF39616, EZF60773, KDB31349, EZF50140, KDB31349, EZG14423, EZG14424, EZG03853, EZG03854, EZF92843, EZF92844, EZF82099, EZF82100
	SUB 5	XM_003231368, XM_047750547, XM_047750548, EGD91985, KFL62981, AY344482, KFL62982, KDB38286, KDB38287, KDB38288, EZG21395, EZG21396, EZG21397, EZF57181, EZG10904, EZF89119, EZG10905, EZG10906, EZF99878, EZF99879, EZF99880, EZF67748, EZF67749, EZF67750, EZF46431, EZF46432, EZF46433, EZF27492, EZF27493, EZF27494
	SUB 6	EGD86731, KDB32624, EZG15666, EZF94094, EZG05369, EZF62064, EZF83437, EZF51383, EZF40766, EZF20202
	SUB 7	KDB35176, EZG18361, EZF64675, EZF85911, EZF54006, EZF43438, EZF24398
T. mentagrophytes	SUB 2	MN549281, MN385834
	SUB 3	EZF14297, KF146901, MN549287, MN549283
	SUB 6	KF146902, MN549285, MN385835
	SUB 7	KF146903
T. benhamiae	SUB 1	XM_003016607, DAA78824, AY437858, EFE36008
	SUB 2	AY437853, XM_003012189, DAA74736, EFE31595
	SUB 3	AY437854, XM_003013110, DAA75608, EFE32516

	SUB 4	AY437855, XM_003012735, DAA75245, EFE32141
	SUB 5	AY437856, XM_003011623, DAA74213, EFE31029
	SUB 6	XM_003016967, DAA79165, EFE36368, AY437857
	SUB 7	EFE35120, DAA78018, AY437852
	SUB 8	DAA75355
	SUB 9	DAA72645
	SUB 10	DAA77632
	SUB 11	DAA78049
	SUB 12	DAA77583
T. tonsurans	SUB 1	FJ348236
	SUB 2	OP727793, OP727792, OP727791, OP727790, OP727789, FJ348237
	SUB 3	OP727773, OP727772, OP727771, OP727770, OP727769, FJ348238
	SUB 4	FJ348239
	SUB 5	OP727798, OP727797, OP727796, OP727795, OP727794, FJ348240
	SUB 7	EZF24398, FJ348241
T. interdigitale	SUB 1	KDB24046, EZF36350
	SUB 2	KDB20915, EZF30374
	SUB 3	KDB23788, EZF35472
	SUB 4	KDB20509, EZF31175
	SUB 5	KAG8212329, KAG5217218, KAG5205231, KDB27873, EZF30715
	SUB 6	KDB24326, EZF32878
	SUB 7	KDB26008, EZF36275
T. verrucosum	SUB 1	AY439105
	SUB 2	AY439106
	SUB 3	AY439107
	SUB 4	AY439108
	SUB 5	AY439109

	SUB 6	AY439110
	SUB 7	AY439111
M. canis	SUB 1	AJ431178
	SUB 2	AJ431179
	SUB 3	ON755069, ON755068, ON755067, ON755066, ON755065, ON755064, AJ431180
T. soudanense	SUB 1	EZF72351, EZF72352
	SUB 2	EZF78605
	SUB 3	EZF71682
	SUB 4	EZF71610, EZF71611
	SUB 5	EZF78423
	SUB 6	EZF72657
	SUB 7	EZF75321
T. equinum	SUB 1	EGE09552, FJ356722
	SUB 2	FJ356723
	SUB 3	FJ356724
	SUB 4	EU076571
	SUB 5	FJ356725
	SUB 6	FJ356726
	SUB 7	EU076572
E. floccosum	SUB 2	MN216337
	SUB 3	MN206114
	SUB 6	MN177931
T. violaceum	SUB 2	OAL69919
A.uncinatum	SUB 11	XM_033552916, KAF3480152
