# Supplementary material for: Diagnostic ability of Peptidase S8 gene in the Arthrodermataceae causing dermatophytoses: A metadata analysis
Source: PLoS One. 2024 Jul 9;19(7):e0306829. doi: 10.1371/journal.pone.0306829 (PMC11232979; doi:10.1371/journal.pone.0306829)
Supplement: S2 Table — (PDF) [file pone.0306829.s002.pdf]

Supplementary Table 2: Estimation of statistical significance in the computed phylogenetic distance (*p* value) of different subtilisin subtypes

Primary Subtilisin Subtype	Comparison Subtilisin Subtype	*p* value based on t-test
SUB1	SUB2	0.000192
SUB1	SUB3	0.021514
SUB1	SUB4	0.089645
SUB1	SUB5	0.000145
SUB1	SUB6	0.044066
SUB1	SUB7	0.066027
SUB2	SUB1	0.000192
SUB2	SUB3	0.000372
SUB2	SUB4	0.001964
SUB2	SUB5	0.040563
SUB2	SUB6	0.000193
SUB2	SUB7	0.000196
SUB3	SUB4	0.275828
SUB3	SUB5	0.000228
SUB3	SUB6	0.310015
SUB3	SUB7	0.229873
SUB3	SUB1	0.021514
SUB3	SUB2	0.000372
SUB4	SUB5	0.001302
SUB4	SUB6	0.416511
SUB4	SUB7	0.5
SUB4	SUB1	0.089645
SUB4	SUB2	0.001964
SUB4	SUB3	0.275828
SUB5	SUB6	0.000128

SUB5	SUB7	0.000133
SUB5	SUB1	0.000145
SUB5	SUB2	0.040563
SUB5	SUB3	0.000228
SUB5	SUB4	0.001302
SUB6	SUB7	0.397345
SUB6	SUB1	0.044066
SUB6	SUB2	0.000193
SUB6	SUB3	0.310015
SUB6	SUB4	0.416511
SUB6	SUB5	0.000128
SUB7	SUB1	0.066027
SUB7	SUB2	0.000196
SUB7	SUB3	0.229873
SUB7	SUB4	0.5
SUB7	SUB5	0.000133
SUB7	SUB6	0.397345
