# Supplementary material for: Diagnostic ability of Peptidase S8 gene in the Arthrodermataceae causing dermatophytoses: A metadata analysis
Source: PLoS One. 2024 Jul 9;19(7):e0306829. doi: 10.1371/journal.pone.0306829 (PMC11232979; doi:10.1371/journal.pone.0306829)

Supplementary Figure 1: PCR targeting ITS region and Subtilisin region for *Penicillium* species isolated from environment

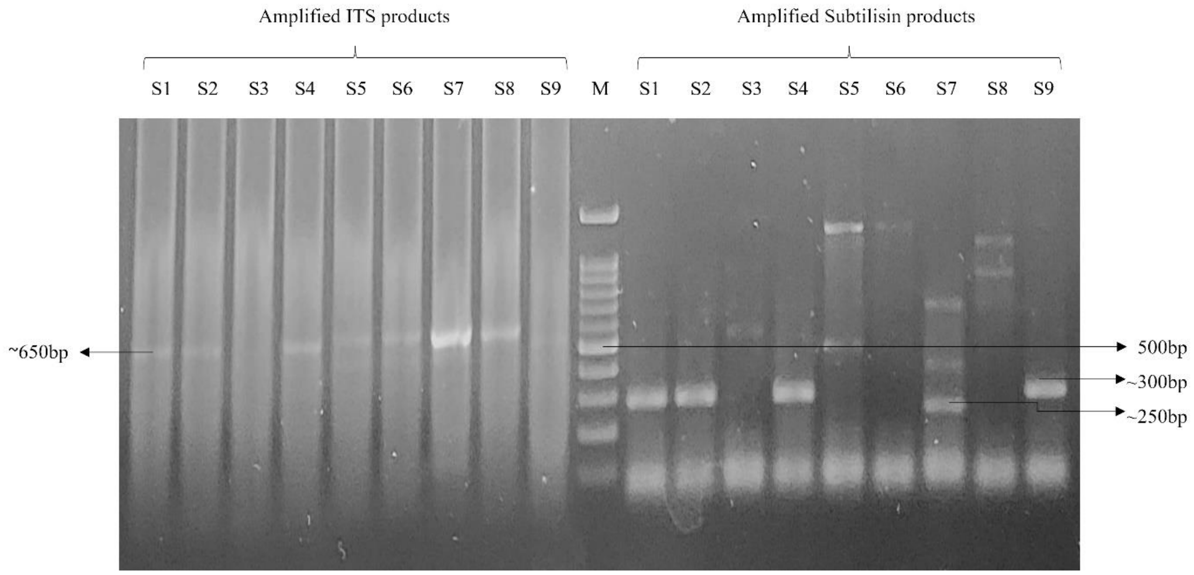

Supplement: S1 Fig — (PDF) [file pone.0306829.s003.pdf]
