# Supplementary material for: Diagnostic ability of Peptidase S8 gene in the Arthrodermataceae causing dermatophytoses: A metadata analysis
Source: PLoS One. 2024 Jul 9;19(7):e0306829. doi: 10.1371/journal.pone.0306829 (PMC11232979; doi:10.1371/journal.pone.0306829)

Supplementary Figure 2: Phylogenetic tree for the alkaline protease/uncharacterized protein of *Penicillium* species available in NCBI in comparison with the subtilisin of Arthrodermataceae species

.

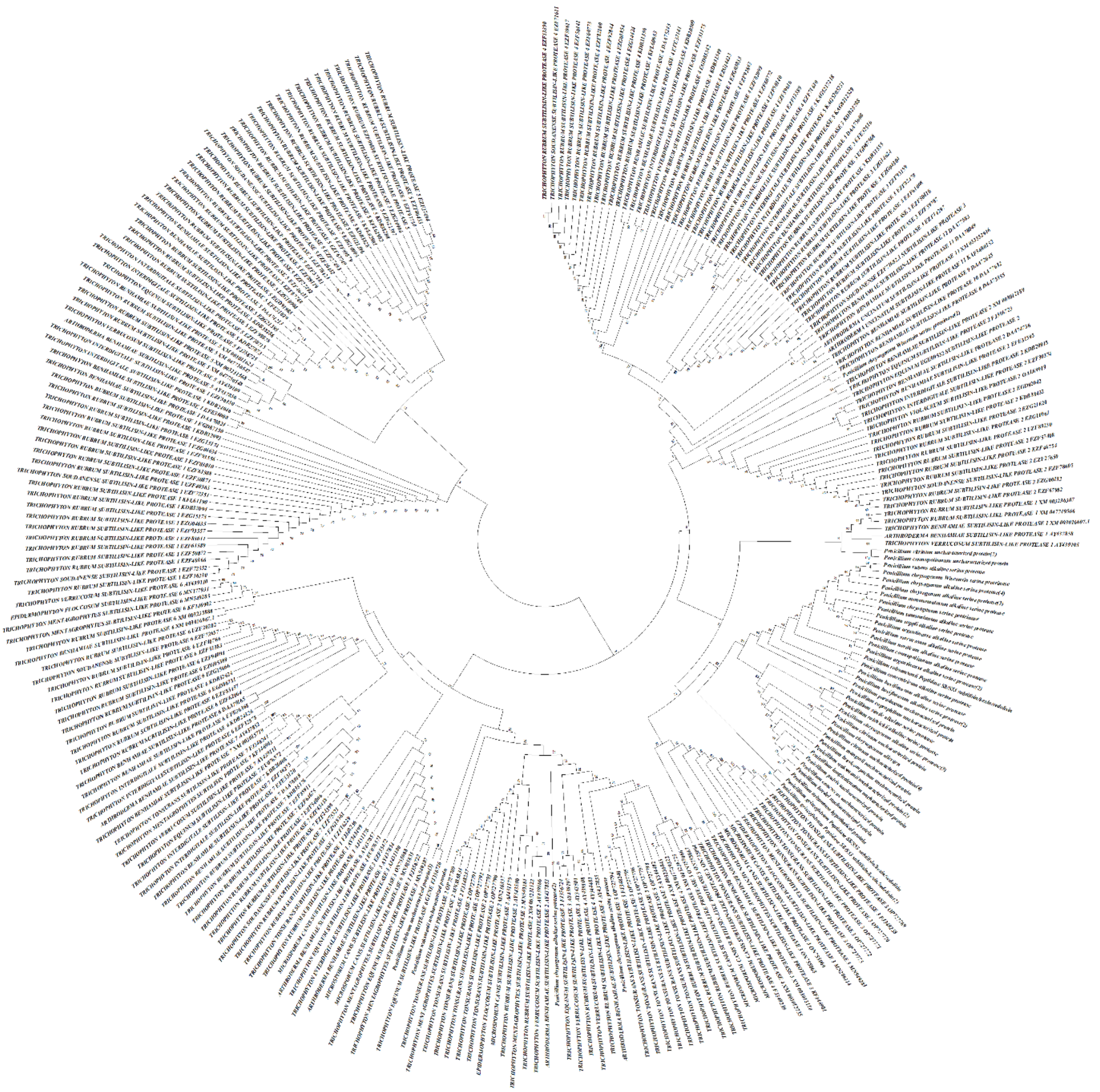

Supplement: S2 Fig — (PDF) [file pone.0306829.s004.pdf]
