# Supplementary material for: Diagnostic ability of Peptidase S8 gene in the Arthrodermataceae causing dermatophytoses: A metadata analysis
Source: PLoS One. 2024 Jul 9;19(7):e0306829. doi: 10.1371/journal.pone.0306829 (PMC11232979; doi:10.1371/journal.pone.0306829)
Supplement: S1 File — (PDF) [file pone.0306829.s005.pdf]

Supplementary Sequence List 1: Sequences of 16 isolates used for phylogenetic tree construct (Figure 5) which are not uploaded to NCBI nucleotide database

>T. mentagrophytes complex sample23

AACGATGGAATGAGCGACAAGGACCTCGACTGCCACCCCTCCTGGAATTACGGGCACCCACCGCC
GCCGTTTGATTCGTCGTGGCGCCAAAGCCATGGGTGGCATGAAGTACACCTACTACTTCCCAACTG
GCCTCAAGGGATACTCTGGTCACTTCGATGAGCAGATGATCAAGGAGATTTCCAAGCGCGGGTGA
TGTAGGCATCAATTATGTCTCCCTAAGAGTGAGGCCCATCCACTCACAGTCTTTCTAGGTCAAATA
CAGTTGACGCGTGATGTCCGCGTCCAAATCAACGCTATTGAGCAGGATGACAACGTTGTCATCCTG
GGGTCTTGCCCGTATCTGCACCCAGGAGCCAGGCGGCACCACCTACAACTATGACAACTCTGCTGT
CAAGGGCACCACTGCCTACGTGATTGACACCGGTACCGATTTCCCGCACGAGGAATTCGACGGTC
GCCGTGCCACCTGGGGTGAGAACTTCGTTGATGACATGGACATGGACTGCAATGTCCATGTTACTC
ACGTCTCCGGAACCGTTGGTGCCAGGACCTTCGGTGTTGCTAAGAAGAGCAACATCGATGCCGTG
ACGGTCCTTGACTGCGACGTGTCTGCTTACTACTCTGGTG

>T. mentagrophytes complex sample24
ATGGGCGGCATGAAGTACACCTACTAGTTCCCAACTGGCCTGAAGGGATACTCTGGTCACTTCGAT
GAGCAGATGATCAAGGAGATTTCCAAGCGCGGTGATGTAGGCCTCAATTATGTCTCGCTAAGAGT
GAGCCCCATCAACTCACAGTCTTTCTAGGTCAAATACGGTTGAGCGTGATGTCCGCGTCCAAATCA
ACGCTATTGAGCAGGATGACAACGTTCCATCCTGGGGTCTTGCCCGTGTCTGCTCCCAGGAGCCCG
GCGGCACCACCTACAACTATGACAACTCTGCTGGCGAGGGCACCACTGCCTACGTTATTGACACCG
GTACCGATTTCCAGCACGAGGAATTCGACGGAGAGCCGTGCCACCTGGGGTGAGAACTTCGTTGA
TGACATGGACATGGACTGCGATGTCCATGGTACTCACGTCTCCGGAACCGTTGGTGCCAGGACCTT
CGGTGTTGCTAAGAAGAGCAACATCGATGCCGTAAAGGTCCTTGACTGCGACGTTGCTGGTTCCTA
CTCTGGTGTG

>T. mentagrophytes complex sample25
TAGTACACCTACTACTTCTCAACTGGCCTCAAGGGATACTCTGGTCACTTCGATGAGCAGATGATC
AAGGAGATTTCCAAGCGCGGTGATGTAGGCCTCAATTATGTCTCCCTAAGAGTGAGCCCCATCAAC
TCACAGTCTTTCTAGGTCAAATACATTGCAGCGTGATGTCCGCGTCCAAATCAACGCTATTGAGCA
GGATGACAACGTTTCATCCTGGGGTCTTGCCCGTGTCGGCTCCCAGGAGCCCGGTGGCACCACCTA
CAACTATGACAACTCTGCTGGGAAGGGCACCACTGCCTACGTTATTGACACCGGTACCGATATCCC
GCACGAGGAATTCGACGGAGGCCGTGCCACCTGGGGTGAGAACTTCGTTGATGACATGGACATGG
ACTGCAATGTCCATGGTACTCACGTCTCCGGAACCGTTGGTGCCAGGACCTTCAGTGTTGCTAAGA
AGAGCGACATCGTTGCCGTGAAGGTCCTTGACTGCGACGTTTCTGGCTACTACTCTGGTGTCACC

>T. mentagrophytes complex sample26
AAGCCATGGGTGGCATGAAGTACACCTACAACTTCCCAACTGGCCTCAAGGGATACTCTGGTCACT
TCGATGAGCAGATGATCAAGGAGATTTCCAAGCGCGCTGATGTACGCATCAATTATGTCTCCCTAA
GAGTGAGCACCATCAACTCACAGTCTTTCTAGGTCAAATACATTGAGCGTGATGTCCGCGTCCAAA
TCAACGCTATTGAGCAGCAGGACAACGTTCCATCCTGGGGTCTTGCCCGTGTCGGCTCCCAGGAGC
CCGGTGGCACCACCTACTACTATGACAGCACTGCTGGCGAGGGCACCACTGCCTACATTATTGACA
CCGGTACCGATATCCAGCACGAGGAATTCGACGGAGGCCGTGCCACCTGGGGTGAGAACTTCGTT
GATGACATGGACATGGACTGCAATGGCCATGGTACTCACGTCTCCGGAACCGTTGGTGGCAGGAC
CTTCGGTGTTGCTAAGAAGAGCAACATCGTTGCCGTGAAGGTCCTTGACTGCGACGTGTCTGCCTC
CTACTCTGGTGTC

>T. mentagrophytes complex sample27
TCCCACCGCTCCTGGGTTAACCGCACCCACCGCCGCCGTTTGATTCGTCGTGGCGCCAAAGCCATG
GGTGGCATGAAGTACACCTACAACTTCCCAACTGGCCTCAAGGGATACTCTGGTCACTTCGATGAG
CAGATGATCAAGGAGATTTCCAAGCGCGCTGATGTAGGCCTCATTTATGTCTCCCTAAGAGTGAGG
CCCATCAACTCACAGTCTTTCTAGGTCAAATACATTGCCGCGTGATGTGCCCGCGTCCAAATCAAC
GCTATTGAGCAGCAGGACAACGTTCCATCCTGGGGTCTTGCCCGTGTCGGCTCCCAGGAGCCCGGT

GGCACCACCTACTACTATGACAGCACTGCTGGCGAAGGCACCACTGCCTACATTATTGACACCGGT
ACCGATATCCAGCACGAGGAATTCGACGGAGGCCGTGCCACCTGGGGTGAGAACTTCGTTGATGA
CATGGACATGGACTGCAATGGCCATGGTACTCACGTCTCCGGAACCGTTGGTGGCAGGACCTTCGG
TGTTGCTAAGAAGAGC

>T. mentagrophytes complex sample28
CAAGGACTTCGACTCCCACCGCTCCTGGGTTAACCGCACCCGCCGCCGCCGTTTGATTCGTCGTGG
AGTACGCGTGGGTGGCATGTAGTACACCTACAAGTTCCCAACTGGCCTCAAGGGATACTCTGGTCA
CTTCGATGAGCAGATGATCAAGGAGATTTCCAAGCGCGCTGATGTATGCATCAATTATGTCTCCCT
AAGAGTGAGCACCATCAACTCACAGTCTTTCTAGGTCAAATACATTGAGCGTGATGCCCGCGTCCA
AATCAACGCTATTGAGCAGCATGACAACGTTCCATCCTGGGGTCTTGCCCGTGTCGGCTCCCAGGA
GCCCGGCGGCACCACCTACAACTATGACAACTCTGCTGGGAAGGGCACCACTGCCTACGTTATTGA
CACCGGTACCGATTTCCAGCACGAGGAATTCGACGGAGGCCGTGCCACCTGGGGTGAGAACTTCG
TTGATGACATGGACATGGACTGCGATGTCCATGTTACTCACGTCTCCGGAACCGTTGGTGCCAGGA
CCTTCGGTGTTGCTAAGAAGAGCAACATCGATGCCGTAAAGGTCCTTGACTGCGACGTTGCTGGTT
ACTACTCTGGTG

>T. mentagrophytes complex sample29
CCGCTCCTGGGTTAACCGCACCCACCGCCGCCGTTTGATTCGTCGTGGCGCCAAAGCCATGGGTGG
CATGAAGTACACCTACAACTTCCCAACTGGCCTCAAGGGATACTCTGGTCACTTCGATGAGCAGAT
GATCAAGGAGATTTCCAAGCGCGCTGATGTACGCGTATAAAAGGTCTTCCCAAATCTGCCCTCTCA
CTCAGAGAGGTTCTAGGACAAATACATTGAGCGTGATGCCCGCGTCCAAATCAACGCTATTGAGC
AGCAGGACAACGTTCCATCCTGGGGTCTTGCCCGTGTCGGCTCCCAGGAGCCCGGTGGCACCACCT
ACTACTATGACAGCACTGCTGGCGAAGGCACCACTGCCTACATTATTGACACCGGTACCGATATCC
AGCACGAGGAATTCGACGGAGGCCGTGCCACCTGGGGTGAGAACTTCGTTGATGACATGGACATG
GACTGCAATGGCCATGGTACTCACGTCTCCGGAACCGTTGGTGGCAGGACCTTCGGTGTTGCTAAG
AAGAGCAACATCGTTGCCGTGAAGGTCCTTGACTGCAACGGGTCTGGCTCCAACTCTGGTGTCATC
ATGGGTATGCAATGGGCTACCGAGGATGCCCAGAGCAAGGGTGCCGACAAGGCCGTCGTCA

>T. mentagrophytes complex sample30
TAAACCATGGGTGGCATGAAGTACACCTACAACTTCCCAACTGGCCTCAAGGGATACTCTGGTCAC
TTCGATGAGCAGATGATCAAGGAGATTTCCAAGCGCGCTGATGTACGCATCAATTATGTCTCCCTA
AGAGTGAGCACCATCAACTCACAGTCTTTCTAGGTCAAATACATTGAGCGTGATGTCCGCGTCCAA
ATCAACGCTATTGAGCAGCAGGACAACGTTCCATCCTGGGGTCTTGCCCGTGTCGGCTCCCAGGAG
CCCGGTGGCACCACCTACTACTATGACAGCACTGCTGGCGAGGGCACCACTGCCTACATTATTGAC
ACCGGTACCGATATCCAGCACGAGGAATTCGACGGAGGCCGTGCCACCTGGGGTGAGAACTTCGT
TGATGACATGGACATGGACTGCAATGACCATGGTACTCACGTCTCCGGAACCGTTGGTGGCAGGA
CCTTCGGTGTTGCTAAGAAGAGCAACATCGTTGCCGTGAAGGTCCTTGACTGC

>T. mentagrophytes complex sample31
ATGGGCGGCATGAAGTACACCTACTAGTTCCCAACTGGCCTGAAGGGATACTCTGGTCACTTCGAT
GAGCAGATGATCAAGGAGATTTCCAAGCGCGGTGATGTAGGCCTCAATTATGTCTCGCTAAGAGT
GAGCCCCATCAACTCACAGTCTTTCTAGGTCAAATACGGTTGAGCGTGATGTCCGCGTCCAAATCA
ACGCTATTGAGCAGGATGACAACGTTCCATCCTGGGGTCTTGCCCGTGTCTGCTCCCAGGAGCCCG
GCGGCACCACCTACAACTATGACAACTCTGCTGGCGAGGGCACCACTGCCTACGTTATTGACACCG
GTACCGATTTCCAGCACGAGGAATTCGACGGAGAGCCGTGCCACCTGGGGTGAGAACTTCGTTGA
TGACATGGACATGGACTGCGATGTCCATGGTACTCACGTCTCCGGAACCGTTGGTGCCAGGACCTT
CGGTGTTGCTAAGAAGAGCAACATCGATGCCGTAAAGGTCCTTGACTGCGACGTTGCTGGTTCCTA
CTCTGGTGTG

>T. mentagrophytes complex sample32
AACGATGGAATGAGCGACAAGGACCTCGACTGCCACCCCTCCTGGAATTACGGGCACCCACCGCC
GCCGTTTGATTCGTCGTGGCGCCAAAGCCATGGGTGGCATGAAGTACACCTACTACTTCCCAACTG
GCCTCAAGGGATACTCTGGTCACTTCGATGAGCAGATGATCAAGGAGATTTCCAAGCGCGGGTGA

TGTAGGCATCAATTATGTCTCCCTAAGAGTGAGGCCCATCCACTCACAGTCTTTCTAGGTCAAATA
CAGTTGACGCGTGATGTCCGCGTCCAAATCAACGCTATTGAGCAGGATGACAACGTTGTCATCCTG
GGGTCTTGCCCGTATCTGCACCCAGGAGCCAGGCGGCACCACCTACAACTATGACAACTCTGCTGT
CAAGGGCACCACTGCCTACGTGATTGACACCGGTACCGATTTCCCGCACGAGGAATTCGACGGTC
GCCGTGCCACCTGGGGTGAGAACTTCGTTGATGACATGGACATGGACTGCAATGTCCATGTTACTC
ACGTCTCCGGAACCGTTGGTGCCAGGACCTTCGGTGTTGCTAAGAAGAGCAACATCGATGCCGTG
ACGGTCCTTGACTGCGACGTGTCTGCTTACTACTCTGGTG

>T. mentagrophytes complex sample33
CAAGGACTTCGACTCCCACCGCTCCTGGGTTAACCGCACCCGCCGCCGCCGTTTGATTCGTCGTGG
AGTACGCGTGGGTGGCATGTAGTACACCTACAAGTTCCCAACTGGCCTCAAGGGATACTCTGGTCA
CTTCGATGAGCAGATGATCAAGGAGATTTCCAAGCGCGCTGATGTATGCATCAATTATGTCTCCCT
AAGAGTGAGCACCATCAACTCACAGTCTTTCTAGGTCAAATACATTGAGCGTGATGCCCGCGTCCA
AATCAACGCTATTGAGCAGCATGACAACGTTCCATCCTGGGGTCTTGCCCGTGTCGGCTCCCAGGA
GCCCGGCGGCACCACCTACAACTATGACAACTCTGCTGGGAAGGGCACCACTGCCTACGTTATTGA
CACCGGTACCGATTTCCAGCACGAGGAATTCGACGGAGGCCGTGCCACCTGGGGTGAGAACTTCG
TTGATGACATGGACATGGACTGCGATGTCCATGTTACTCACGTCTCCGGAACCGTTGGTGCCAGGA
CCTTCGGTGTTGCTAAGAAGAGCAACATCGATGCCGTAAAGGTCCTTGACTGCGACGTTGCTGGTT
ACTACTCTGGTG

>T. mentagrophytes complex sample34
TCCCACCGCTCCTGGGTTAACCGCACCCACCGCCGCCGTTTGATTCGTCGTGGCGCCAAAGCCATG
GGTGGCATGAAGTACACCTACAACTTCCCAACTGGCCTCAAGGGATACTCTGGTCACTTCGATGAG
CAGATGATCAAGGAGATTTCCAAGCGCGCTGATGTAGGCCTCATTTATGTCTCCCTAAGAGTGAGG
CCCATCAACTCACAGTCTTTCTAGGTCAAATACATTGCCGCGTGATGTGCCCGCGTCCAAATCAAC
GCTATTGAGCAGCAGGACAACGTTCCATCCTGGGGTCTTGCCCGTGTCGGCTCCCAGGAGCCCGGT
GGCACCACCTACTACTATGACAGCACTGCTGGCGAAGGCACCACTGCCTACATTATTGACACCGGT
ACCGATATCCAGCACGAGGAATTCGACGGAGGCCGTGCCACCTGGGGTGAGAACTTCGTTGATGA
CATGGACATGGACTGCAATGGCCATGGTACTCACGTCTCCGGAACCGTTGGTGGCAGGACCTTCGG
TGTTGCTAAGAAGAGC

>T. mentagrophytes complex sample35
AAGCCATGGGTGGCATGAAGTACACCTACAACTTCCCAACTGGCCTCAAGGGATACTCTGGTCACT
TCGATGAGCAGATGATCAAGGAGATTTCCAAGCGCGCTGATGTACGCATCAATTATGTCTCCCTAA
GAGTGAGCACCATCAACTCACAGTCTTTCTAGGTCAAATACATTGAGCGTGATGTCCGCGTCCAAA
TCAACGCTATTGAGCAGCAGGACAACGTTCCATCCTGGGGTCTTGCCCGTGTCGGCTCCCAGGAGC
CCGGTGGCACCACCTACTACTATGACAGCACTGCTGGCGAGGGCACCACTGCCTACATTATTGACA
CCGGTACCGATATCCAGCACGAGGAATTCGACGGAGGCCGTGCCACCTGGGGTGAGAACTTCGTT
GATGACATGGACATGGACTGCAATGGCCATGGTACTCACGTCTCCGGAACCGTTGGTGGCAGGAC
CTTCGGTGTTGCTAAGAAGAGCAACATCGTTGCCGTGAAGGTCCTTGACTGCGACGTGTCTGCCTC
CTACTCTGGTGTC

>T. mentagrophytes complex sample36
TAGTACACCTACTACTTCTCAACTGGCCTCAAGGGATACTCTGGTCACTTCGATGAGCAGATGATC
AAGGAGATTTCCAAGCGCGGTGATGTAGGCCTCAATTATGTCTCCCTAAGAGTGAGCCCCATCAAC
TCACAGTCTTTCTAGGTCAAATACATTGCAGCGTGATGTCCGCGTCCAAATCAACGCTATTGAGCA
GGATGACAACGTTTCATCCTGGGGTCTTGCCCGTGTCGGCTCCCAGGAGCCCGGTGGCACCACCTA
CAACTATGACAACTCTGCTGGGAAGGGCACCACTGCCTACGTTATTGACACCGGTACCGATATCCC
GCACGAGGAATTCGACGGAGGCCGTGCCACCTGGGGTGAGAACTTCGTTGATGACATGGACATGG
ACTGCAATGTCCATGGTACTCACGTCTCCGGAACCGTTGGTGCCAGGACCTTCAGTGTTGCTAAGA
AGAGCGACATCGTTGCCGTGAAGGTCCTTGACTGCGACGTTTCTGGCTACTACTCTGGTGTCACC

>T. mentagrophytes complex sample37

CCGCTCCTGGGTTAACCGCACCCACCGCCGCCGTTTGATTCGTCGTGGCGCCAAAGCCATGGGTGG
CATGAAGTACACCTACAACTTCCCAACTGGCCTCAAGGGATACTCTGGTCACTTCGATGAGCAGAT
GATCAAGGAGATTTCCAAGCGCGCTGATGTACGCGTATAAAAGGTCTTCCCAAATCTGCCCTCTCA
CTCAGAGAGGTTCTAGGACAAATACATTGAGCGTGATGCCCGCGTCCAAATCAACGCTATTGAGC
AGCAGGACAACGTTCCATCCTGGGGTCTTGCCCGTGTCGGCTCCCAGGAGCCCGGTGGCACCACCT
ACTACTATGACAGCACTGCTGGCGAAGGCACCACTGCCTACATTATTGACACCGGTACCGATATCC
AGCACGAGGAATTCGACGGAGGCCGTGCCACCTGGGGTGAGAACTTCGTTGATGACATGGACATG
GACTGCAATGGCCATGGTACTCACGTCTCCGGAACCGTTGGTGGCAGGACCTTCGGTGTTGCTAAG
AAGAGCAACATCGTTGCCGTGAAGGTCCTTGACTGCAACGGGTCTGGCTCCAACTCTGGTGTCATC
ATGGGTATGCAATGGGCTACCGAGGATGCCCAGAGCAAGGGTGCCGACAAGGCCGTCGTCA

>T. mentagrophytes complex sample38
TAAACCATGGGTGGCATGAAGTACACCTACAACTTCCCAACTGGCCTCAAGGGATACTCTGGTCAC
TTCGATGAGCAGATGATCAAGGAGATTTCCAAGCGCGCTGATGTACGCATCAATTATGTCTCCCTA
AGAGTGAGCACCATCAACTCACAGTCTTTCTAGGTCAAATACATTGAGCGTGATGTCCGCGTCCAA
ATCAACGCTATTGAGCAGCAGGACAACGTTCCATCCTGGGGTCTTGCCCGTGTCGGCTCCCAGGAG
CCCGGTGGCACCACCTACTACTATGACAGCACTGCTGGCGAGGGCACCACTGCCTACATTATTGAC
ACCGGTACCGATATCCAGCACGAGGAATTCGACGGAGGCCGTGCCACCTGGGGTGAGAACTTCGT
TGATGACATGGACATGGACTGCAATGACCATGGTACTCACGTCTCCGGAACCGTTGGTGGCAGGA
CCTTCGGTGTTGCTAAGAAGAGCAACATCGTTGCCGTGAAGGTCCTTGACTGC
