# Supplementary material for: Diagnostic ability of Peptidase S8 gene in the Arthrodermataceae causing dermatophytoses: A metadata analysis
Source: PLoS One. 2024 Jul 9;19(7):e0306829. doi: 10.1371/journal.pone.0306829 (PMC11232979; doi:10.1371/journal.pone.0306829)
Supplement: S2 File — (PDF) [file pone.0306829.s006.pdf]

Supplementary Sequence list 2: Sequences obtained from amplification of eight *Penicillium* isolates using the primers designed in this study

>Penicillium sp sample 1
CATGATGACACTCAACTTCCAAGAAATCAATTTTCCTAATCTCAGCACCAGTCAGCGGAAGCCCGC
TCTTTTGACAAACCTGCCCAAGTACCCTTGGAACCATAGCGAGAAGTACTGGATCAATGCAAGGCT
ATGCGACAATCTCTACCGGCCCCAATTCGCTCGAAATGACATTCTTGGGAAGCTTTGCATCGAGAA
TATCGACTTTGAGCCTCGATGGAGGAATATA

> Penicillium sp sample 2
AAGCCCGCTCTTTTGACAAACCTGCCCAAGTACCCTTGGAACCATAGCGAGAAGTACTGGATCAAT
GCAAGGCTATGCGACAATCTCTACCGGCCCCAATTCGCTCGAAATGACATTCTTGGGAAGCTTTGC
ATCGAGAATATCGACTTTGAGCCTCGATGGAGGAATATAATTCGCGCGGACGACCACCCATGGAT
TCGACAACATCGTGTTCATGACAGCAA

> Penicillium sp sample 4
AAGCCCGCTCTTTTGACAAACCTGCCCAAGTACCCTTGGAACCATAGCGAGAAGTACTGGATCAAT
GCAAGGCTATGCGACAATCTCTACCGGCCCCAATTCGCTCGAAATGACATTCTTGGGAAGCTTTGC
ATCGAGAATATCGACTTTGAGCCTCGATGGAGGAATATAATTCGCGCGGACGACCACCCATGGAT
TCGACAACATCGTGTTCATGACAGCAA

> Penicillium sp sample 5
CATGATGACACTCAACTTCCAAGAAATCAATTTTCCTAATCTCAGCACCAGTCAGCGGAAGCCCGC
TCTTTTGACAAACCTGCCCAAGTACCCTTGGAACCATAGCGAGAAGTACTGGATCAATGCAAGGCT
ATGCGACAATCTCTACCGGCCCCAATTCGCTCGAAATGACATTCTTGGGAAGCTTTGCATCGAGAA
TATCGACTTTGAGCCTCGATGGAGGAATATA

> Penicillium sp sample 6
CATGATGACACTCAACTTCCAAGAAATCAATTTTCCTAATCTCAGCACCAGTCAGCGGAAGCCCGC
TCTTTTGACAAACCTGCCCAAGTACCCTTGGAACCATAGCGAGAAGTACTGGATCAATGCAAGGCT
ATGCGACAATCTCTACCGGCCCCAATTCGCTCGAAATGACATTCTTGGGAAGCTTTGCATCGAGAA
TATCGACTTTGAGCCTCGATGGAGGAATAT

> Penicillium sp sample 7
GAAGCCCGCTCTTTTGACAAACCTGCCCAAGTACCCTTGGAACCATAGCGAGAAGTACTGGATCA
ATGCAAGGCTATGCGACAATCTCTACCGGCCCCAATTCGCTCGAAATGACATTCTTGGGAAGCTTT
GCATCGAGAATATCGACTTTGAGCCTCGATGGAGGAATATAATTCGCGCGGACGACCACCCATGG
ATTCGACAACATCGTGTTCATGACAGCAA

> Penicillium sp sample 8
CATGATGACACTCAACTTCCAAGAAATCAATTTTCCTAATCTCAGCACCAGTCAGCGGAAGCCCGC
TCTTTTGACAAACCTGCCCAAGTACCCTTGGAACCATAGCGAGAAGTACTGGATCAATGCAAGGCT
ATGCGACAATCTCTACCGGCCCCAATTCGCTCGAAATGACATTCTTGGGAAGCTTTGCATCGAGAA
TATCGACTTTGAGCCTCGATGGAGGAATATAA

> Penicillium sp sample 9
AAGCCCGCTCTTTTGACAAACCTGCCCAAGTACCCTTGGAACCATAGCGAGAAGTACTGGATCAAT
GCAAGGCTATGCGACAATCTCTACCGGCCCCAATTCGCTCGAAATGACATTCTTGGGAAGCTTTGC
ATCGAGAATATCGACTTTGAGCCTCGATGGAGGAATATAATTCGCGCGGACGACCACCCATGGAT
TCGACAACATCGTGTTCATGACAGCAA

*Penicillium sp sample 3 did not yield any amplification with the SUB7 primers
