# Supplementary material for: Diagnostic ability of Peptidase S8 gene in the Arthrodermataceae causing dermatophytoses: A metadata analysis
Source: PLoS One. 2024 Jul 9;19(7):e0306829. doi: 10.1371/journal.pone.0306829 (PMC11232979; doi:10.1371/journal.pone.0306829)
Supplement: Attachment — Submitted filename: Response to reviewers_June2024.docx [file pone.0306829.s008.docx]

**Response to reviewers**

Reviewer #1

**Comment 1**

Abstract

Lines 2-3

Instead of ,,...etiological agents of dermatophytosis...” should be ,,...etiological agents of dermatophytoses” (plural)

**Response:** We are grateful to the reviewer for the insights provided and accept the correction.

Revised manuscript Abstract Line 3 “...etiological agents of dermatophytoses”

**Comment 2**:

Introduction

Lines 7-9

To make it sound more professional and appropriate, I recommend rewording: ,, While based on direct microscopic examination of skin scrapings, the presence of fungal elements like conidia and true hyphae indicate fungal infections, the diagnosis is not conclusive and identification requires other culture-dependent or culture-independent methods.”

**Response**: Sentence rephrased based on the inputs provided.

**Comment 3:**

Results

Line 164

In case of ,,NCBI Nucleotide database” a capital letter in case of word ,,nucleotide” is not necessary.

**Response:** Typographical errors are corrected in the revised manuscript.

**Comment 4 and 5**:

Figure 1 and Figure 2a

The colors assigned in the legend to individual components of the pie chart are invisible, which makes it difficult for a potential reader to know which markings correspond to the data shown on the pie chart. The resolution of Figure 1 should also be improved.

**Response:** The colours assigned in the legend to individual components of the pie chart have been enlarged for better visibility. The resolution of all the images in the revised submission is 400 dpi .

**Comment 6:**

Figure 3 and 5

The resolution should also be improved.

**Response:** The resolution of all images in the revised submission is 400 dpi .

**Comment 7:**

Discussion

Line 232

To the best of my knowledge should be ,,...causative agents of dermatophytoses...” instead of ,,...causative agents of dermatophytosis...”

**Response:** We are grateful to the reviewer for the insights provided and accept the correction.

Revised manuscript Discussion Line 232 : “...causative agents of dermatophytosis...”
